# Improved clinical outcomes in advanced hepatocellular carcinoma treated with transarterial chemoembolization plus atezolizumab and bevacizumab: a bicentric retrospective study

**DOI:** 10.1186/s12885-023-11389-x

**Published:** 2023-09-18

**Authors:** Fei Cao, Changsheng Shi, Guofu Zhang, Jun Luo, Jiaping Zheng, Weiyuan Hao

**Affiliations:** 1 Institute of Basic Medicine and Cancer (IBMC), The Cancer Hospital of the University of Chinese Academy of Sciences (Zhejiang Cancer Hospital), Chinese Academy of Sciences, Hangzhou, Zhejiang 310022 China; 2 Department of Interventional, The Third Affiliated Hospital of Wenzhou Medical University, Ruian, 325200 Zhejiang People’s Republic of China; 3 Zhejiang Elderly Care Hospital, Hangzhou, Zhejiang 310022 China

**Keywords:** Hepatocellular carcinoma, Atezolizumab, Bevacizumab, Overall survival, Progression-free survival

## Abstract

**Purpose:**

The aim of the present study was to assess the efficacy and safety of transarterial chemoembolization (TACE) combined with atezolizumab and bevacizumab (hereafter, TACE-Atez/Bev) in the treatment of advanced hepatocellular carcinoma (HCC) patients.

**Materials and methods:**

Clinical information was collected from consecutive patients with advanced HCC who received treatment with TACE-Atez/Bev or Atez/Bev from April 2021 and October 2022. Treatment response, overall survival (OS), and progression-free survival (PFS) were the primary outcomes of this study. Adverse events (AEs) were the secondary outcomes. Propensity score matching (PSM) analysis was applied to reduce bias between two groups.

**Results:**

This study included 62 patients in the TACE-Atez/Bev group and 77 patients in the Atez/Bev group. The objective response rate (ORR) of the TACE-Atez/Bev group and the Atez/Bev group were 38.7% and 16.9% (*P*=0.004). However, there was no statistical difference in disease control rate between the two groups (69.4% vs 63.6%, *P*=0.479). Before PSM, the median OS was 14 months in the TACE-Atez/Bev group and 10 months in the Atez/Bev group (*P*=0.014). The median PFS in the TACE-Atez/Bev and Atez/Bev groups was 10 months and 6 months, respectively (*P*=0.001). After PSM, the median OS in the two groups was 14 months and 9 months, respectively (*P*=0.01). The median PFS was 7 months and 6 months, respectively (*P*=0.036). Multivariable analysis showed that treatment method was independent prognostic factors affecting OS.

**Conclusions:**

Compared with Atez/Bev treatment, TACE-Atez/Bev showed better OS, PFS, and ORR for Chinese patients with advanced HCC, with an acceptable safety profile.

**Supplementary Information:**

The online version contains supplementary material available at 10.1186/s12885-023-11389-x.

## Introduction

Hepatocellular carcinoma (HCC) is one of the most common malignancies worldwide [1, 2], and although ultrasound and serum alpha-fetoprotein (AFP) levels are monitored in high-risk populations for early detection of HCC, most patients are diagnosed with advanced stage, which seriously affects the prognosis of patients with HCC [3–5]. Both the IMbrave150 trial and ORIENT-32 trials demonstrated that immune checkpoint inhibitors (ICIs) combined with anti-angiogenesis therapy significantly improved patient outcomes compared with sorafenib in advanced HCC patients who had not received systemic therapy [6, 7], and atezolizumab combined with bevacizumab (Atez/Bev) was recommended as the first-line therapy for HCC [8]. However, 20.3% of HCC patients showed progressive disease (PD) after treatment with Atez/Bev [6]. Therefore, exploring other therapeutic modalities in combination with antiangiogenic therapy and ICIs may further improve the outcome of HCC patients.

Transarterial chemoembolization (TACE) can improve the survival rate of unresectable HCC patients by inducing tumor avascular necrosis [9, 10]. TACE induced hypoxia response has been reported to promote the release of pro-angiogenic cytokines, leading to tumor angiogenesis [11]. Thus, TACE in combination with anti-angiogenic therapy such as sorafenib or apatinib was shown to be superior to monotherapy [12–14]. In addition, TACE may increase the number of intratumoral CD8 + T cells and transform the immunosuppressive microenvironment into an immune-supporting environment to enhance the response to PD-(L)1 inhibitors [15–17]. Zhu et al. ‘s findings indicated that TACE plus ICIs and anti-angiogenic therapy can significantly improve outcomes in Chinese patients with advanced HCC with an acceptable safety profile compared to TACE monotherapy [16]. Similarly, Huang et al. ‘s study also showed that TACE can improve the efficacy of ICIs combined with anti-angiogenic therapy in the treatment of advanced HCC patients [17].

Therefore, a trimodal approach combining ICIs with anti-angiogenic therapy and TACE may offer an innovative and interesting therapeutic strategy for the treatment of HCC. However, as far as we know, TACE combined with Atez/Bev (TACE-Atez/Bev) for advanced HCC is rarely reported. Hence, the purpose of this study was to retrospectively compare the efficacy and safety of TACE-Atez/Bev treatment with Atez/Bev treatment alone in advanced HCC patients.

## Methods

### Patients

In this retrospective study, 139 advanced HCC patients received TACE-Atez/Bev or Atez/Bev treatment at the Center Hospital of the University of Chinese Academy of Sciences and The Third Affiliated Hospital of Wenzhou Medical University From April 2021 and October 2022.

Patients were included when they met the following criteria: (1) HCC patients older than 18 years of age; (2) Child-Pugh A or B stage; (3) Eastern Cooperative Oncology Group (ECOG) scores 0 or 1. Patients will be excluded when they meet the following criteria: (1) patients with main portal vein obstruction; (2) patients had been treated with anti-angiogenesis therapy, ICIs, or TACE; (3) hepatic dysfunction or renal impairment; (4) in addition to TACE, patients received other treatments such as radiofrequential ablation during this study; (5) loss to follow up.

The present study was carried out in accordance with the principles of the Declaration of Helsinki. The institutional review board of the Center Hospital of the University of Chinese Academy of Sciences and The Third Affiliated Hospital of Wenzhou Medical University approved the present study. Obtain written informed consent from all patients prior to treatment.

### TACE therapy

The femoral artery was punctured using the Seldinger technique, and a 5-F Yashiro catheter (Terumo, Tokyo, Japan) or a 2.7-F microcatheter (Progreat, Terumo, Tokyo, Japan) was was placed in the supply vessels of tumors. Then, 5–20 ml lipiodol and 20–60 mg epirubicin were mixed into the emulsion and slowly injected into the tumor. In this study, all enrolled patients were treated with epirubicin mixed with lipiodol. In addition, appropriate amount of gelatin sponge (100–300 μm or 300–500 μm, Alicon, Hangzhou, China) was injected to supplement embolization. Embolization was performed under fluoroscopic guidance until there was stasis of arterial flow. Hepatic artery angiography was then subsequently performed to confirm sucess of the embolization procedure. For bilobar or huge lesions, at least two TACE sessions 4–6 weeks apart were required to perform complete embolization. TACE was performed in an average of 3.3 ± 2.6 times per patient during the therapy. TACE was not considered if one of the following situations occurred: (1) Child-Pugh C stage (uncontrolled ascites, severe jaundice, significant hepatic encephalopathy, or hepatorenal syndrome); (2) ECOG scores > 2; (3) the target lesions continued to progress after three TACE sessions.

### Atezolizumab/bevacizumab

Atezolizumab and bevacizumab were administered 3–5 days after TACE, once every 3 weeks, at the minimum clinically recommended dose. If patients had adverse event (AEs), the treatment is symptomatic, and if serious AEs occurred, the medication were interrupted or discontinued.

### Definition and evaluation of data

Treatment response, overall survival (OS), and progression-free survival (PFS) were the primary outcomes of this study. Adverse events (AEs) were the secondary outcomes. OS was defined as the time from the patient’s initial treatment to the patient’s death or the end of follow-up. PFS was defined as the time from initial treatment to tumor progression, patient’s death, or the end of follow-up. One month after the initial TACE, patients underwent CT/MRI to assess tumor response (according to Modified Response Evaluation Criteria in Solid Tumors [mRECIST]). Objective response rate (ORR) included complete response (CR) and partial response (PR), while disease control rate (DCR) included CR, PR and stable disease (SD). AEs were recorded and assessed by Common Terminology Criteria for Adverse Events (CTCAE, Version 5.0).

### Follow-up

The present study was followed up until July 31, 2023. If imaging one month after the initial TACE confirms the presence of a viable liver tumor and the patient’s liver function is good, TACE was performed again. Follow-up was completed if the patient died.

### Statistical analyses

SPSS software (version 26.0) and R (version 4.0.3) software was applied to statistical analyses of this study. Independent sample t-test and Chi-squared test were applied to analyze differences between the two groups. Survival and PFS curves were calculated for both groups by using Kaplan-Meier method. Univariate analyses were implemented with the log-rank test, in which variables with P less than 0.1 were entered into the multivariate analyses, which were implemented with the Cox proportional hazard regression model. Propensity score matching (PSM) was performed using a 1:1 nearest neighbor matching procedure with a caliper width set at 0.1 of the SD of the logit of the propensity score. All statistical tests were two tailed, and *P* < 0.05 was considered statistically significant.

## Results

### Study population and patient characteristics

A total of 173 patients were treated with TACE-Atez/Bev or Atez/Bev between April 2021 and October 2022, of which 34 patients were excluded. Finally, 139 eligible patients were enrolled in this study, including 62 patients in the TACE-Atez/Bev group and 77 patients in the Atez/Bev group. After PSM, 61 patients in each group were enrolled. The detailed baseline characteristics of patients in the two groups were presented in Table 1 and Supplementary Table 1.

**Table 1:** Baseline characteristics

Characteristics	Atez/Bev group (*N* = 77) (No, %; Mean ± SD)	TACE-Atez/Bev group (*N* = 62) (No, %; Mean ± SD)	*P* value
**Gender**			0.930
Male	65 (84.4%)	52 (83.9%)	
Female	12 (15.6%)	10 (16.1%)	
**Age (years)**	52.8 ± 11.0	55.8 ± 11.2	0.108
**Hepatitis**			0.449
Hepatitis B	59 (76.6%)	44 (71.0%)	
Other	18 (23.4%)	18 (29.0%)	
**Child-Pugh score**			0.832
A	51 (66.2%)	40 (64.5%)	
B	26 (33.8%)	22 (35.5%)	
**TB (µmol/L)**	19.8 ± 11.2	18.3 ± 9.4	0.422
**Albumin (g/L)**	35.8 ± 4.8	34.6 ± 3.4	0.103
**PT(s)**	14.3 ± 1.2	14.1 ± 1.5	0.560
**AST (µmol/L)**	60.7 ± 47.2	66.8 ± 50.0	0.459
**ALT (µmol/L)**	43.4 ± 24.5	43.2 ± 24.2	0.961
**PLR**	128.9 ± 63.3	146.4 ± 63.5	0.108
**NLR**	3.0 ± 2.0	3.5 ± 1.8	0.090
**Tumor size (cm)**	8.9 ± 3.5	8.4 ± 4.6	0.457
**Tumor number**			0.861
≤ 3	12 (15.6%)	9 (14.5%)	
>3	65 (84.4%)	53 (85.5%)	
**α-Fetoprotein level**			0.569
>400 ng/mL	36 (46.7%)	32 (51.6%)	
≤ 400 ng/ml	41 (53.2%)	30 (48.4%)	
**ECOG**			0.421
0	32 (41.6%)	30 (48.4%)	
1	45 (58.4%)	32 (51.6%)	
**Vascular invasion**			0.906
Absent	34 (44.2%)	28 (45.2%)	
Present	43 (55.8%)	34 (54.8%)	
**Extrahepatic spread**			0.538
Absent	32 (41.6%)	29 (46.8%)	
Present	45 (58.4%)	33 (53.2%)	
**Ascites**			0.114
Absent	46 (59.7%)	45 (72.6%)	
Present	31 (40.3%)	17 (27.4%)	

*Atez/Bev *Atezolizumab/bevacizumab, *TACE *Transarterial chemoembolization, *SD *Standard deviation, *BCLC *Barcelona Clinical Liver Cancer, *TB *Total bilirubin, *PT *Prothrombin time, *AST *Aspartate aminotransferase, *ALT *Alanine aminotransferase, *PLR *Platelet-to-lymphocyte ratio, *NLR *Neutrophil-to-lymphocyte ratio, *ECOG *Eastern Cooperative Oncology Group

The median follow-up period was 13 months (range, 3–25 months) for patients in the TACE-Atez/Bev group and 9 months (range, 3–21 months) for patients in the Atez/Bev group. By the end of follow-up (July 2023), 50 patients (80.6%) in the TACE-Atez/Bev group died, while 63 patients (81.8%) in the Atez/Bev group died.

### Treatment response

In the TACE-Atez/Bev group, there were 1 (1.6%) patients with CR, 23 patients (37.1%) with PR and 19 patients (30.6%) with SD. In the Atez/Bev group, there were 1 (1.3%) patients with CR, 12 patients (15.6%) with PR and 36 patients (46.8%) with SD. Hence, ORR of the TACE-Atez/Bev group and the Atez/Bev group were 38.7% and 16.9%, respectively, showing a statistically significant difference between the two groups (*P* = 0.004). Meanwhile, the DCR of the two groups was 69.4% and 63.6% respectively, which had no significant statistical difference (*P* = 0.479).

### Overall survival

**Fig. 1:**
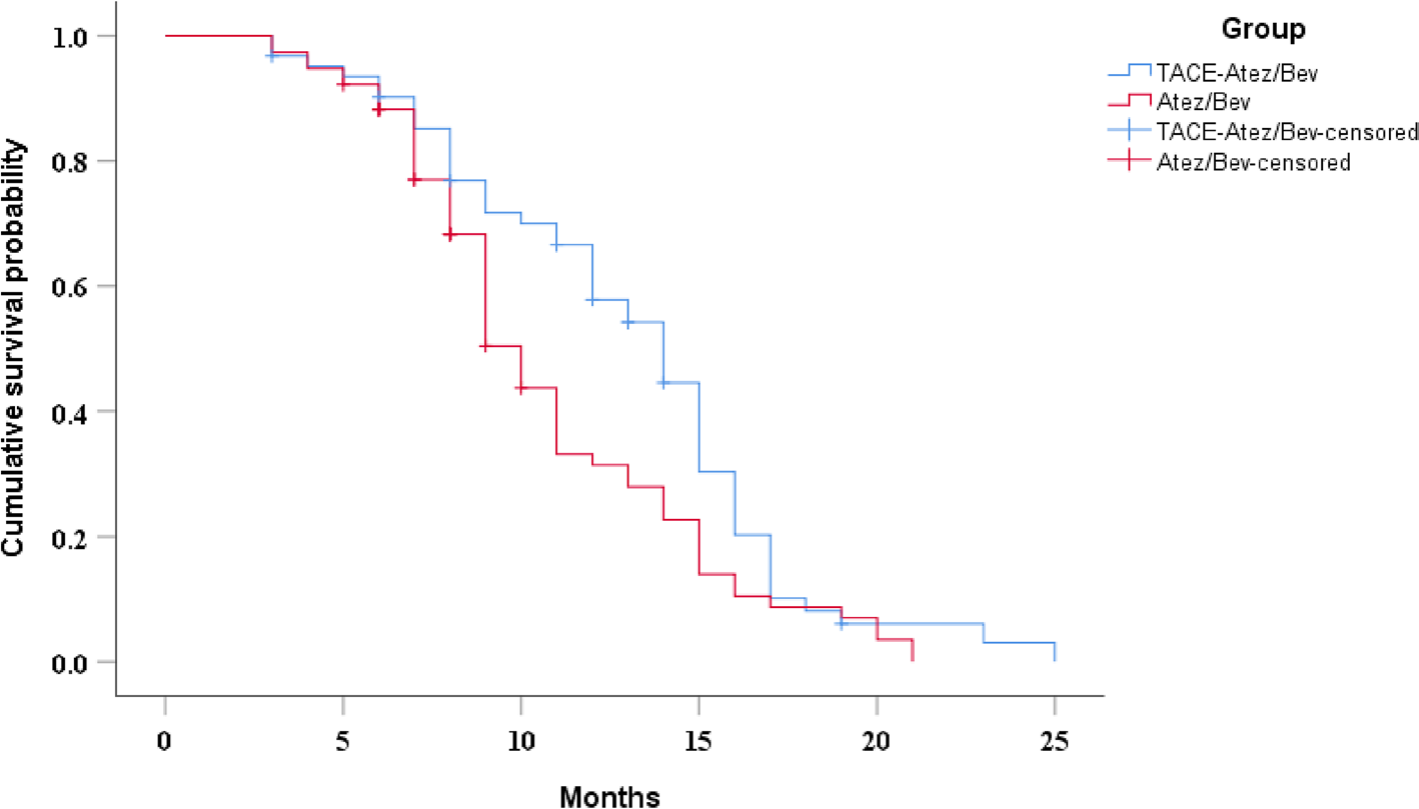
Kaplan-Meier curves of cumulative survival in advanced HCC patients who received TACE-Atez/Bev or Atez/Bev. Kaplan-Meier method and log-rank test were performed to evaluate the differences in OS between the two groups

The median OS was 14 months (95%CI: 12.0–16.0 months) in the TACE-Atez/Bev group and 10 months (95%CI: 9.0–11.0 months) in the Atez/Bev group, and there was a statistically significant difference between the two groups (*P* = 0.014) (Fig. 1). Univariate analyses demonstrated that Hepatitis B, alanine aminotransferase and treatment method were related to OS (Table 2). Multivariate analyses showed that treatment method was independent prognostic factors affecting OS (Table 3). After PSM, the median OS in the two groups was 14 months (95%CI: 12.9–15.1 months) and 9 months (95%CI: 7.9–10.1 months), respectively (*P* = 0.01) (Supplementary Fig. 1).

**Table 2:** Univariate analysis of prognostic factors for overall survival and progression-free survival

Variables	OS		PFS	
	HR (95% CI)	*P* value	HR (95% CI)	*P* value
**Gender**				
Male	1		1	
Female	1.109 (0.676, 1.820)	0.682	1.108 (0.686, 1.790)	0.676
**Age (years)**	1.009 (0.993, 1.026)	0.254	1.009 (0.994, 1.025)	0.249
**Hepatitis**				
Hepatitis B	1		1	
Other	0.690 (0.449, 1.059)	0.089	0.689 (0.454, 1.046)	0.080
**Child-Pugh score**				
A	1			
B	1.143 (0.770, 1.696)	0.509	1.001 (0.686, 1.460)	0.997
**TB (µmol/L)**	1.009 (0.988, 1.030)	0.417	1.014 (0.995, 1.033)	0.165
**Albumin (g/L)**	1.021 (0.978, 1.066)	0.345	1.002 (0.961, 1.045)	0.932
**PT (s)**	0.977 (0.827, 1.154)	0.785	1.102 (0.942, 1.291)	0.226
**AST (µmol/L)**	0.997 (0.993, 1.001)	0.173	0.997 (0.994, 1.001)	0.177
**ALT (µmol/L)**	0.993 (0.985, 1.001)	0.072	0.995 (0.988, 1.002)	0.192
**PLR**	1.002 (1.000, 1.005)	0.083	1.002 (1.000, 1.005)	0.083
**NLR**	1.037 (0.946, 1.136)	0.443	1.051 (0.957, 1.155)	0.301
**Tumor size**	1.016 (0.969, 1.065)	0.510	1.109 (0.975, 1.064)	0.404
**Tumor number**				
≤3	1		1	
≤ 3	0.991 (0.610, 1.611)	0.972	0.780 (0.482, 1.261)	0.311
**α-Fetoprotein level**				
≥ 400 ng/mL	1		1	
≤400 ng/ml	1.113 (0.768, 1.613)	0.573	0.979 (0.688, 1.394)	0.907
**ECOG**				
1	1		1	
0	0.853 (0.589, 1.234)	0.398	0.776 (0.543, 1.109)	0.164
**Vascular invasion**				
Present	1		1	
Absent	1.151 (0.795, 1.666)	0.455	1.144 (0.802, 1.632)	0.457
**Extrahepatic spread**				
Present	1		1	
Absent	1.141 (0.788, 1.653)	0.485	0.869 (0.610, 1.240)	0.439
**Ascites**				
Present	1		1	
Absent	0.778 (0.520, 1.165)	0.223	0.716 (0.485, 1.056)	0.092
**Treatment method**				
Atez/Bev	1		1	
TACE-Atez/Bev	0.651 (0.447, 0.947)	0.025	0.565 (0.392, 0.815)	0.002

*OS *Overall survival, *PFS *Progression-free survival, *HR *Hazard ratio, *CI *Confidence interval, *SD *Standard deviation, *BCLC *Barcelona Clinical Liver Cancer, *TB *Total bilirubin, *PT *Prothrombin time, *AST *Aspartate aminotransferase, *ALT *Alanine aminotransferase, *PLR *Platelet-to-lymphocyte ratio, *NLR *Neutrophil-to-lymphocyte ratio, *ECOG *Eastern Cooperative Oncology Group, *Atez/Bev *Atezolizumab/bevacizumab, *TACE *Transarterial chemoembolization

**Table 3:** Multivariate analysis of prognostic factors for overall survival

Variables	HR (95% CI)	*P* value
**Hepatitis**		
Hepatitis B	1	
Other	0.706 (0.457, 1.090)	0.116
**ALT (µmol/L)**	0.995 (0.987, 1.003)	0.215
**Treatment method**		
Atez/Bev	1	
TACE-Atez/Bev	0.663 (0.453, 0.969)	0.034

*HR *Hazard ratio, *CI *Confidence interval, *ALT *Alanine aminotransferase, *Atez/Bev *Atezolizumab/bevacizumab, *TACE *Transarterial chemoembolization

### Progression-free survival

**Fig. 2:**
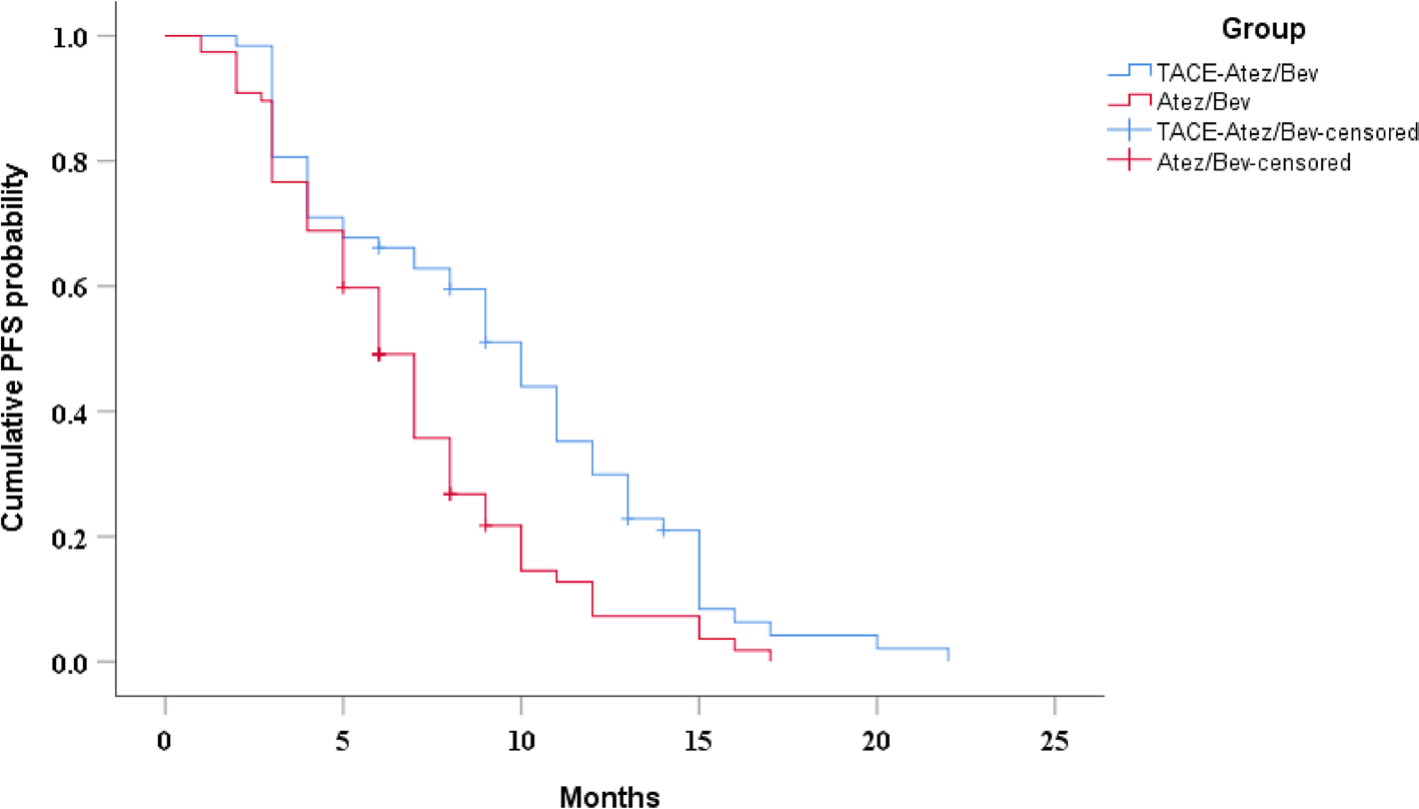
Kaplan-Meier curves of cumulative PFS in advanced HCC patients who received TACE-Atez/Bev or Atez/Bev. Kaplan-Meier method and log-rank test were performed to evaluate the differences in PFS between the two groups

The median PFS in the TACE-Atez/Bev and Atez/Bev groups was 10 (95%CI: 8.4–11.6 months) and 6 months (95%CI: 5.1-7.0 months), respectively, and there was a significant difference between the two groups (*P* = 0.001) (Fig. 2). Univariate analyses demonstrated that Hepatitis B, platelet-to-lymphocyte ratio (PLR), ascites and treatment method were related to PFS (Table 2). Multivariable analysis revealed that PLR, ascites and treatment method were associated with PFS (Table 4). After PSM, the median PFS in the two groups was 7 months (95%CI: 4.5–9.5 months) and 6 months (95%CI: 4.9–7.1 months), respectively (*P* = 0.036) (Supplementary Fig. 2).

**Table 4:** Multivariate analysis of prognostic factors for progression-free survival

Variables	HR (95% CI)	*P* value
**Hepatitis**		
Hepatitis B	1	
Other	0.769 (0.499, 1.186)	0.235
**PLR**	1.003 (1.000, 1.006)	0.031
**Ascites**		
Present	1	
Absent	0.645 (0.434, 0.959)	0.030
**Treatment method**		
Atez/Bev	1	
TACE-Atez/Bev	0.461 (0.311, 0.684)	< 0.001

*HR *Hazard ratio, *CI *Confidence interval, *PLR *Platelet-to-lymphocyte ratio, *Atez/Bev *Atezolizumab/bevacizumab, *TACE *Transarterial chemoembolization

### Safety of combination treatment

A total of 48 patients (77.4%) in the TACE-Atez/Bev group developed pain, fever, nausea and vomiting within 1 week after TACE, and their symptoms were significantly relieved or disappeared after symptomatic treatment. There were no serious AEs associated with TACE, such as liver abscess and biloma. Furthermore, no TACE related deaths occurred.

AEs related to Atez/Bev are shown in Tables 5 and 6. A total of 21 patients (33.9%) in the TACE-Atez/Bev group developed AEs of varying degrees, compared with 24 patients (31.1%) in the Atez/Bev group (*P* = 0.735). Hypertension, proteinuria, fatigue and diarrhea were common AEs in both groups. Meanwhile, 2 patients in the TACE- Atez/Bev group developed gastrointestinal hemorrhage, which was improved by symptomatic supportive treatment and suspension of medication. In addition, 1 patient in the Atez/Bev group developed pneumonia, and the symptom was improved by hormone therapy and drug withdrawal. Meanwhile, no grade 4 or above AEs occurred in this study and no drug-related mortalities occurred.

**Table 5:** Adverse events in the TACE-Atez/Bev group

Adverse Event	All Events	CTCAE Grade		
		1	2	3
**Hypertension**	13 (21.0%)	6 (9.7%)	5 (8.1%)	2 (3.2%)
**Proteinuria**	6 (9.7%)	4 (6.5%)	2 (3.2%)	0 (0%)
**Fatigue**	4 (6.5%)	3 (4.8%)	1 (1.6%)	0(0%)
**Diarrhea**	3 (4.8%)	2 (3.2%)	1 (1.6%)	0 (0%)
**Gastrointestinal hemorrhage**	2 (3.2%)	2 (3.2%)	0 (0%)	0 (0%)

*CTCAE *Common Terminology Criteria for Adverse Events, *Atez/Bev *Atezolizumab/bevacizumab, *TACE *Transarterial chemoembolization

**Table 6:** Adverse events in the Atez/Bev group

Adverse Event	All Events	CTCAE Grade		
		1	2	3
**Hypertension**	15 (19.5%)	9 (11.7%)	4 (5.2%)	2 (2.6%)
**Proteinuria**	7 (9.1%)	3 (3.9%)	3 (3.9%)	1 (1.3%)
**Fatigue**	5 (6.5%)	4 (5.2%)	1 (1.3%)	0 (0%)
**Diarrhea**	3 (3.9%)	2 (2.6%)	1 (1.3%)	0 (0%)
**Pneumonitis**	1 (1.3%)	0 (0%)	1 (1.3%)	0 (0%)

*CTCAE *Common Terminology Criteria for Adverse Events, *Atez/Bev *Atezolizumab/bevacizumab

## Discussion

The combination of TACE and Atez/Bev has the following theoretical advantages [15, 18, 19]: (1) TACE can effectively reduce the intrahepatic tumor burden and promote tumor-specific CD8^+^ T cell response by killing HCC cells and stimulate the exposure of tumor-associated antigens; (2) Bevacizumab can reshape tumor vessels, improve the immune microenvironment caused by hypoxia after TACE, and enhance the efficacy of Atezolizumab. Hence, TACE combined with Atez/Bev may have synergistic and positive effects for the treatment of advanced HCC.

The present study demonstrated that TACE-Atez/Bev had better efficacy in the treatment of advanced HCC, which was mainly manifested as tumor response, median OS and PFS were significantly better than Atez/Bev alone. Finn et al. reported that the median PFS of HCC patients receiving Atez/Bev was 6.8 months [6]. Similarly, the median PFS of patients in Atez/Bev group in this study was 6 months, significantly lower than 10 months in TACE-Atez/Bev group. This may be due to tumor necrosis after TACE, resulting in continuous tumor antigen exposure and enhanced anti-tumor immunity. In addition, studies by Casadei-Gardini and Sinner et al. showed that the median OS of Atez/Bev treatment for advanced HCC was 16.4 months and 16.0 months, respectively [20, 21], which was significantly higher than the median OS of the two groups in this study. Similarly, studies by Maesaka and Persano et al. have shown better survival benefits [22, 23]. All patients in this study were patients with advanced HCC, and most patients had extrhepatic metastases/vascular invasion, which may be the reason for the low median OS in this study.

Two studies conducted by Finn et al. [6] and Persano et al. [24] demonstrated that the ORR of Atez/Bev for advanced HCC patients was 27.3%, which was higher than the ORR after Atez/Bev therapy in this study. Compared with these two studies, all patients in this study had advanced HCC, which may be the reason for the lower ORR. However, patients with Atez/Bev combined with TACE showed a significant increase in ORR. In addition to reducing tumor load, TACE kills HCC cells and causes tumor-associated antigen release, which boosts tumor specific CD8^+^ T-cell responses [15]. Hence, TACE combined with Atez/Bev may significantly improve the efficacy and survival of patients with advanced HCC.

Schobert et al. studied inflammatory biomarkers in HCC patients treated with TACE and found that PLR was associated with tumor response and PFS [25]. Similarly, the results of multivariate analysis in this study showed that PLR was an independent risk factor for PFS. Ascites is an important part of Child puge score of liver function. The results of multivariate analysis demonstrated that ascites is an independent risk factor affecting patients’ PFS. In addition, this study showed that TACE-Atez/Bev was the only independent protective factor affecting patients’ OS and PFS. Therefore, PLR, ascites, and treatment method may be factors affecting prognosis.

Similar to other studies [6, 26, 27], the common AEs associated with Atez/Bev were hypertension, proteinuria, fatigue, diarrhea, etc., and most of them were grade 1 or 2. After symptomatic treatment, these AEs were significantly improved or disappered. Bleeding (including fatal events) is a known AE of bevacizumab. Similar to the Qin study [26], 2 patients in this study had gastrointestinal bleeding, which stopped after temporary drug withdrawal and symptomatic treatment such as stomach protection and acid suppressant. Furthermore, similar to Ren et al.‘s study, one patient in this study developed pneumonia and improved with drug withdrawal and hormone therapy [28]. Meanwhile, there was no significant difference in the incidence of AEs between the two groups, and TACE did not increase the incidence of Atez/Bev-related complications.

Non-randomized design is the major limitations of the present study. Therefore, it is necessary to conduct a multi-center prospective clinical study to further validate the results of this study. In addition, due to the limited sample size, stratified analysis was not conducted in the present study.

## Conlusion

In conclusion, for advanced HCC, compared with Atez/Bev, TACE combined with Atez/Bev indicated clinically significant improvement in OS and PFS. However, further prospective clinical trials with larger samples sizes are needed to improve quality of evidence.

### Supplementary Information

**Additional file 1: Supplementary Table 1. **Baseline Characteristics after propensity score matching. **Supplementary Figure 1. **Kaplan-Meier curves of cumulative survival in advanced HCC patients who received TACE-Atez/Bev or Atez/Bev after PSM. **Supplementary Figure 2. **Kaplan-Meier curves of cumulative PFS in advanced HCC patients who received TACE-Atez/Bev or Atez/Bev after PSM.

## Data Availability

All data that support the findings of this study are collected objectively and are available from the corresponding author on reasonable request.
