# Supplementary material for: Improved clinical outcomes in advanced hepatocellular carcinoma treated with transarterial chemoembolization plus atezolizumab and bevacizumab: a bicentric retrospective study
Source: BMC Cancer. 2023 Sep 18;23:873. doi: 10.1186/s12885-023-11389-x (PMC10506240; doi:10.1186/s12885-023-11389-x)
Supplement: Supplementary file 1 — Additional file 1: Supplementary Table 1. Baseline Characteristics after propensity score matching. Supplementary Figure 1. Kaplan-Meier curves of cumulative survival in advanced HCC patients who received TACE-Atez/Bev or Atez/Bev after PSM. Supplementary Figure 2. Kaplan-Meier curves of cumulative PFS in advanced HCC patients who received TACE-Atez/Bev or Atez/Bev after PSM. [file 12885_2023_11389_MOESM1_ESM.docx]

**Supplementary Table 1 Baseline Characteristics after propensity score matching**

| **Characteristics** | **Atez/Bev group**  (N=61)  (No, %; Mean ± SD) | **TACE-Atez/Bev group** (N=61)  (No, %; Mean ± SD) | ***P* value** |
| --- | --- | --- | --- |
| **Gender** |  |  | 1.000 |
| Male | 51 (83.6%) | 51 (83.6%) |  |
| Female | 10 (16.4%) | 10 (16.4%) |  |
| **Age (years)** | 53.9±11.3 | 55.6±11.2 | 0.391 |
| **Hepatitis** |  |  | 0.410 |
| Hepatitis B | 47 (77.0%) | 43 (70.5%) |  |
| Other | 14 (23.0%) | 18 (29.5%) |  |
| **Child-Pugh score** |  |  | 0.565 |
| A | 42 (68.9%) | 39 (63.9%) |  |
| B | 19 (31.1%) | 22 (36.1%) |  |
| **TB (µmol/L)** | 19.7 ± 11.9 | 18.5±9.4 | 0.541 |
| **Albumin (g/L)** | 36.0 ± 4.9 | 34.5±5.5 | 0.110 |
| **PT(s)** | 14.3 ± 1.1 | 14.1±1.5 | 0.489 |
| **AST (µmol/L)** | 62.0±48.1 | 63.8±44.2 | 0.834 |
| **ALT (µmol/L)** | 44.3±25.2 | 43.4±24.3 | 0.829 |
| **PLR** | 139.6±64.4 | 159.6±85.4 | 0.148 |
| **NLR** | 3.1±1.9 | 3.6±2.0 | 0.167 |
| **Tumor size (cm)** | 9.1±3.5 | 8.3±4.6 | 0.281 |
| **Tumor number** |  |  | 0.592 |
| ≤3 | 7 (11.5%) | 9 (14.8%) |  |
| ＞3 | 54 (88.5%) | 52 (85.2%) |  |
| **α-Fetoprotein level** |  |  | 0.469 |
| ＞400 ng/mL | 28 (45.9%) | 32 (52.5%) |  |
| ≤400 ng/ml | 33 (54.1%) | 29 (47.5%) |  |
| **ECOG** |  |  | 0.717 |
| 0 | 28 (45.9%) | 30 (49.2%) |  |
| 1 | 33 (54.1%) | 31 (50.8%) |  |
| **Vascular** **invasion** |  |  | 0.856 |
| Absent | 29 (47.5%) | 28 (45.9%) |  |
| Present | 32 (52.5%) | 33 (54.1%) |  |
| **Extrahepatic spread** |  |  | 0.466 |
| Absent | 25 (41.0%) | 29 (47.5%) |  |
| Present | 36 (59.0%) | 32 (52.5%) |  |
| **Ascites** |  |  | 0.127 |
| Absent | 36 (59.0%) | 44 (72.1%) |  |
| Present | 29 (41.0%) | 17 (27.9%) |  |

Note. Atez/Bev: atezolizumab/bevacizumab; TACE: transarterial chemoembolization; SD: Standard deviation; BCLC: Barcelona Clinical Liver Cancer; TB: [Total](javascript:;) [bilirubin](javascript:;); PT: Prothrombin time; AST: Aspartate aminotransferase; ALT: Alanine aminotransferase; PLR: Platelet-to-lymphocyte ratio; NLR: Neutrophil-to-lymphocyte ratio; ECOG: Eastern Cooperative Oncology Group.


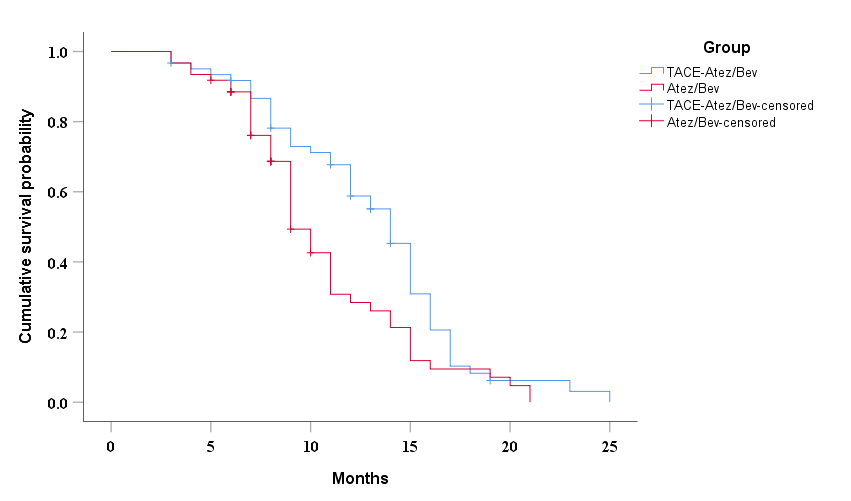


**Supplementary Figure 1**  Kaplan-Meier curves of cumulative survival in advanced HCC patients who received TACE-Atez/Bev or Atez/Bev after PSM.


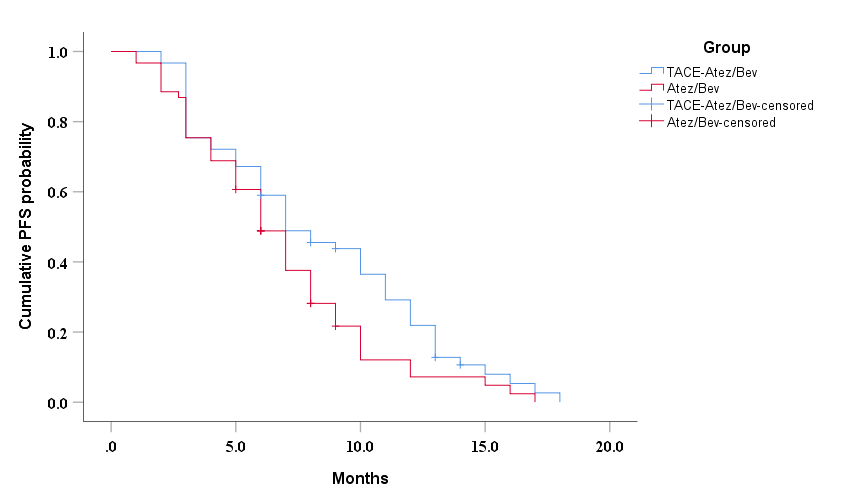


**Supplementary Figure 2**  Kaplan-Meier curves of cumulative PFS in advanced HCC patients who received TACE-Atez/Bev or Atez/Bev after PSM.
